# Supplementary material for: Cloning, Expression, Characterization and Immobilization of a Recombinant Carboxylesterase from the Halophilic Archaeon, Halobacterium salinarum NCR-1
Source: Biomolecules. 2024 Apr 30;14(5):534. doi: 10.3390/biom14050534 (PMC11118615; doi:10.3390/biom14050534)
Supplement: Supplementary file 1 [file biomolecules-14-00534-s001.zip › biomolecules-2912956-supplementary.pdf]

biomolecules

MDPI

Article

Cloning, expression, characterization and immobilization of a recombinant carboxylesterase from the halophilic archaeon, *Halobacterium salinarum* NCR-1

Nestor D. Ortega-de la Rosa[1], Evelyn Romero-Borbón[2], Jorge Alberto Rodríguez[3], Angeles Camacho-Ruiz[4] and Jesús Córdova[2,*]

Supplementary Materials:

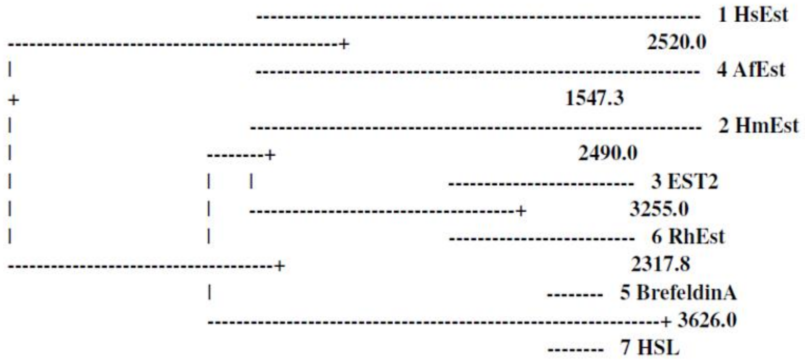

Alignment score = 42402.00
Alignment score per aligned residue pair = 8.98
Sequence identities = 1022
Percent sequence identity = 0.22
Number of sequences = 7
Alignment length = 1080
Number of residues = 2927
Number of gaps = 4633

Figure S1. PRALINE tree representation of alignment of the amino acid sequence of *H. salinarum* esterase (HsEst, GenBank: AAG19778.1) with other characterized proteins of the HSL proteins family. HmEst (NSSF)*: H. marismortui* esterase Lip C (GenBank: AAV45166.1); **EST2** (NSSF)*: Alicyclobacillus acidocaldarius* **esterase (**NCBI Reference Sequence: WP_012812080.1; **PDB:** 1U4N)**; BrefeldinA*:*** *Bacillus subtilis* Brefeldin A esterase (GenBank: AAC12774.1; PDB ID:1JKM), **RhEST** (NSSF)**:** *Rhodococcus* sp. **h**eroin esterase **(**NCBI Reference Sequence: WP_259118130.1; **PDB:** 4RNC); **AfEst** (31.35% Per. Ident.)**:** *Archaeoglobus fulgidus* esterase **(**NCBI Reference Sequence: PDB: 5FRD); and **HSL** (NSSF)**:** hormone sensitive lipase (NCBI Reference Sequence: NP_005348.2). The characterized proteins are marked with (∗). Per. Ident.: Percentage identity with HsEst determined with NCBI protein-protein blast. NSSF: No significant similarity found determined with NCBI protein-protein blast.

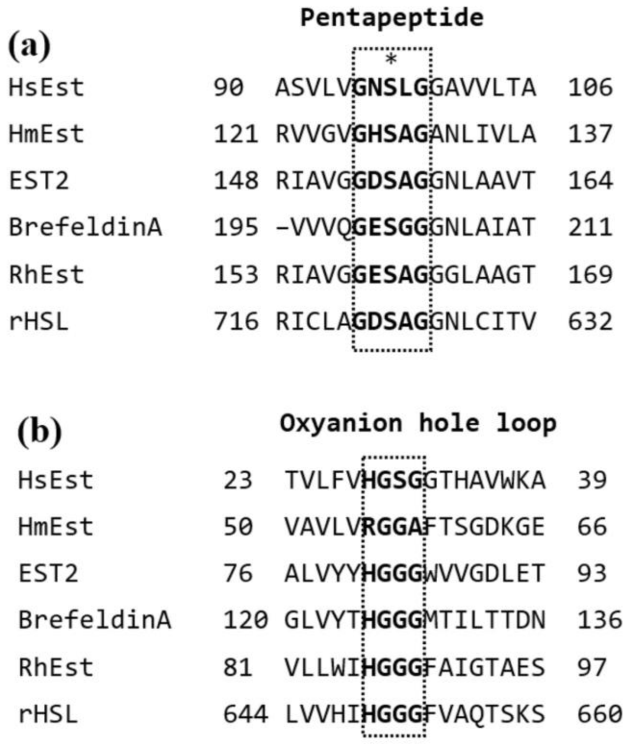

Figure S2. Alignment of the amino acid sequence of HsEST with other proteins of the HSL family showing the pentapeptide containing the active site serine (a) and the oxyanion-hole loop (b). *H. salinarum* esterase (HsEst), *H. marismortui* esterase Lip C (HmEst), esterase from *Alicyclobacillus acidocaldarius* (EST2), *B. subtilis* Brefeldin A esterase (BrefeldinA), *Rhodococcus* sp. heroin esterase (RhEST) and hormone sensitive lipase (rHSL). The residues marked in bold indicate the most conserved residues within the amino acid sequences. The catalytic serine is marked (∗).

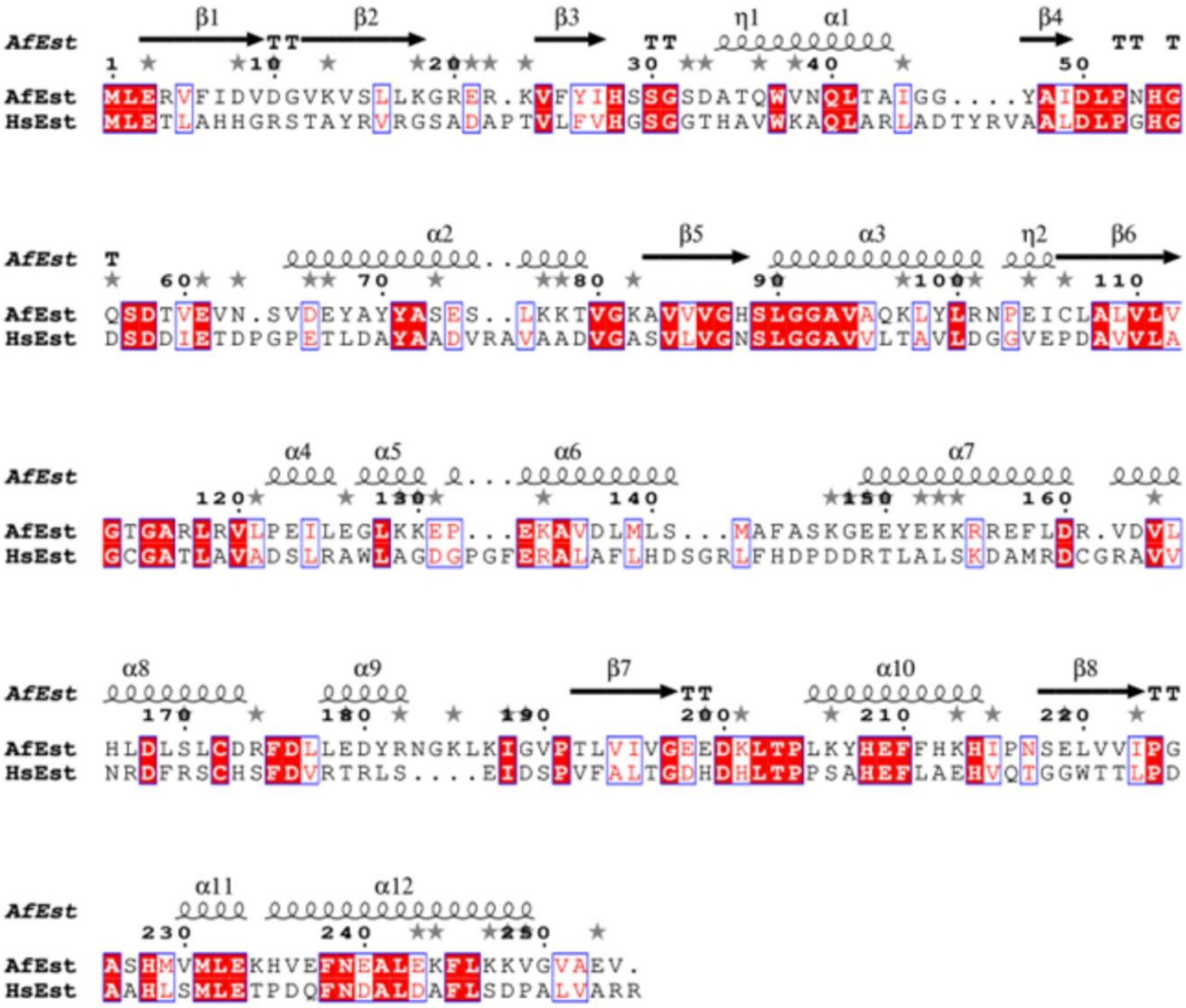

Figure S3. Pairwise sequence alignment with ESPript 3.0 between HsEst and AfEst (PDB: 5FRD) that share 31.25 % identity. The dots in the alignment and in the structural elements indicate gaps. The numbers at the top of sequence for each line correspond to the amino acid position of AfEst. The secondary structure elements of both enzymes are shown above the alignment: alpha-helix (α), beta-strand (β). The conserved amino acids are highlighted in red while the conserved regions are contained within blue lines. Amino acid residues of HsEst (S97, D211 and H239) and AfEst (S89, D200 and H228) involved in the active site are signalled with *.

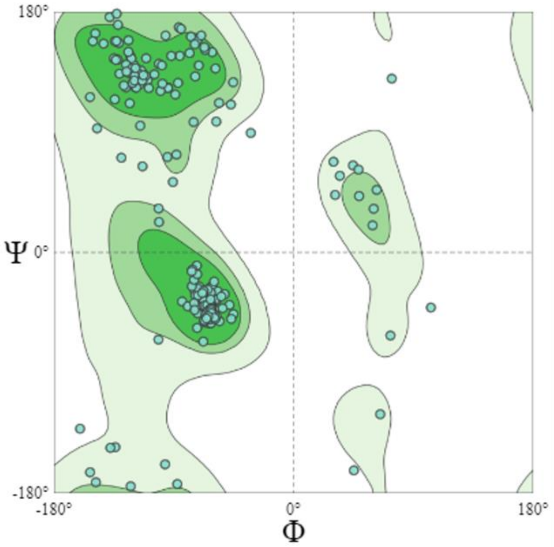

Figure S4. Structure assessment of HsEst model analyzed Molprobity in Swiss model. Ramachandran score 89.77% for HsEst model obtained with Phyre2.

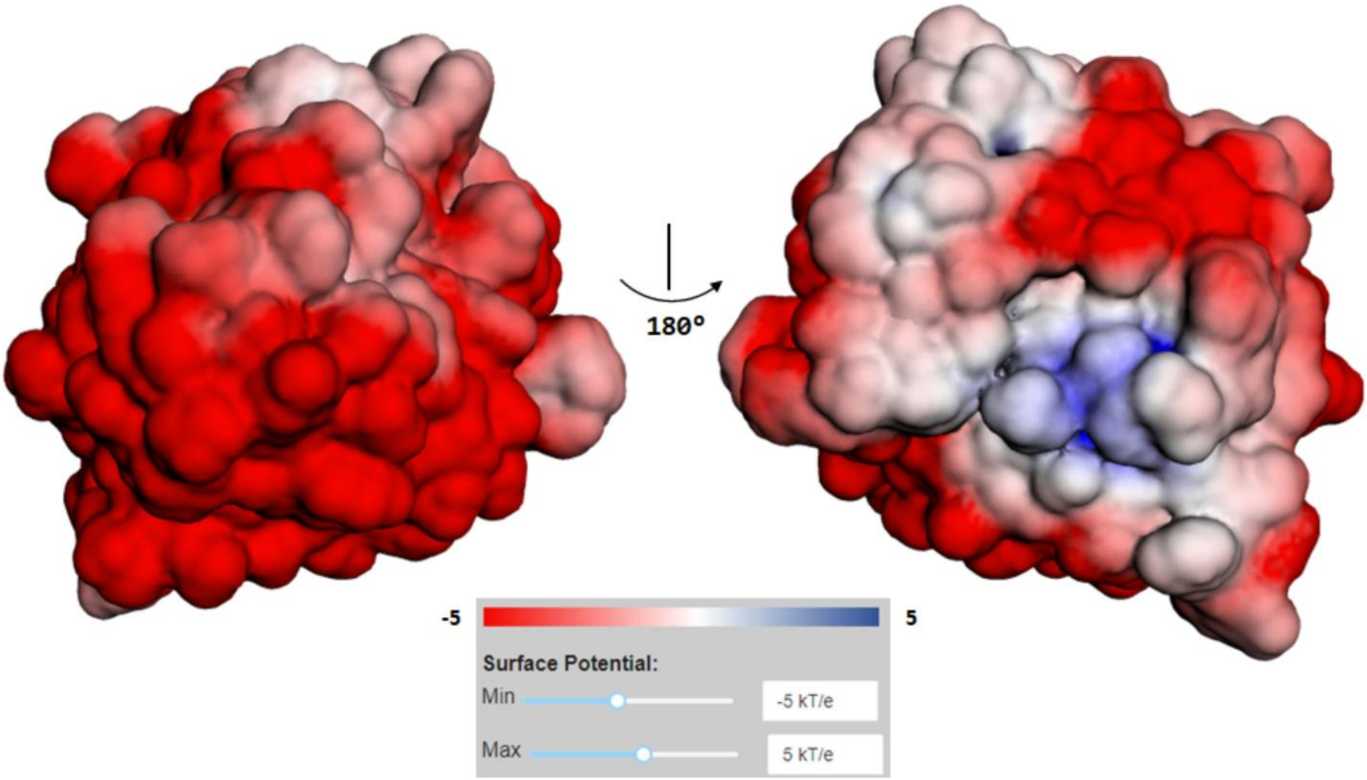

Figure S5. Relative surface electrostatic potentials of HsEst as obtained using PDB2PQR with PARSE forcefield online program. Online visualization with 3Dmol: the red surface corresponding to negatively charged residues (Asp, Glu) and the blue surface corresponding to positively charged residues (Arg, Lys).

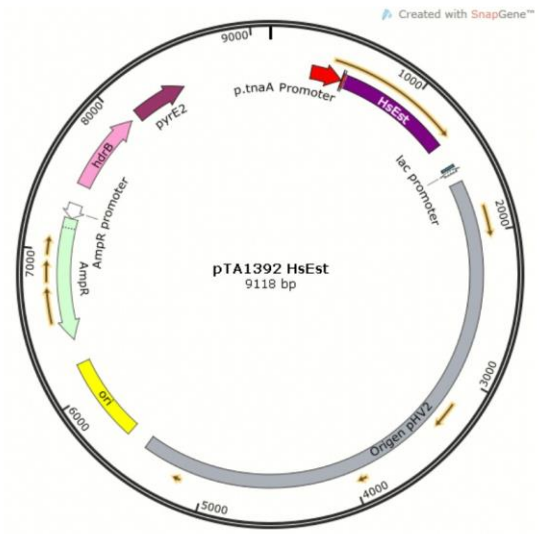

(**a**)

	5′	
517	CACCACCACCACCACCA**ATG**TTGGAGACGCTTGCCCACCACGGACGGTCGACAGCCTACCGCGTCCGTGGGT	589
590	CCGCAGACGCACCCACCGTGTTGTTCGTCCACGGCAGCGGCGGCACCCACGCCGTCTGGAAAGCACAGCTG	659
661	GCACGCCTCGCGGACACGTATCGGGTCGCCGCCCTGGACCTACCGGGGCACGGCGACAGCGACGACATCGA	731
733	AACCGACCCTGGCCCGGAGACGCTTGACGCGTACGCGGCGGACGTGCGCGCCGTCGCCGCCGATGTCGGTG	805
805	CGTCCGTCCTGGTGGGGAACAGCCTCGGCGGTGCGGTCGTGCTCACCGCAGTCCTCGACGGAGGCGTTGAG	876
877	CCGGACGCCGTGGTGCTTGCCGGATGTGGCGCAACGCTCGCGGTCGCCGACTCGCTTCGCGCCTGGCTCGCC	949
9510	GGCGATGGCCCCGGGTTCGAGCGGGCACTGGCGTTCCTCCACGACAGCGGCCGGCTGTTCCACGACCCCGA	1021
1022	CGACCGCACGCTCGCCCTCTCGAAGGACGCAATGCGGGACTGCGGGCGCGCCGTCGTCAACCGCGACTTCC	1094
1094	GGTCGTGTCACAGCTTCGATGTCCGAACGCGACTCTCGGAGATCGACAGCCCGGTGTTCGCGCTCACCGGTG	1166
1167	ATCACGACCACCTCACACCGCCCTCGGCCCACGAGTTCCTCGCGGAACACGTGCAGACAGGGGGGTGGACG	1238
1239	ACGCTCCCGGATGCCGCACACCTCTCGATGCTCGAAACACCCGACCAGTTCAACGACGCCCTCGATGCGTTTC	1312
1313	TCAGTGACCCCGCGCTAGTGGCCCGCCGA**TAG**	1335
	3′	

(**b**)

Figure S6. Schematic representation of the plasmid (pTA1392-HsEst), showing the insertion site of AAG19778.1 gene from *H. salinarum* NRC-1, which encodes a putative carboxylesterase generated with SnapGene™ (**a**). Nucleotide sequence of HsEst in frame contained in plasmid pTA1392, where the start (ATG) and stop (TAG) codons for protein synthesis are indicated in bold, the 6xHis-tag in N-terminus is underlined, in bold and italics the codon for the initial methionine (ATG) of HsEst **(b)**. In (**a**) the numbers close to the circle indicate the nucleotide position for each feature, in (**b**) the numbers on the sides of the sequence indicate the position of the first and last base in each row.

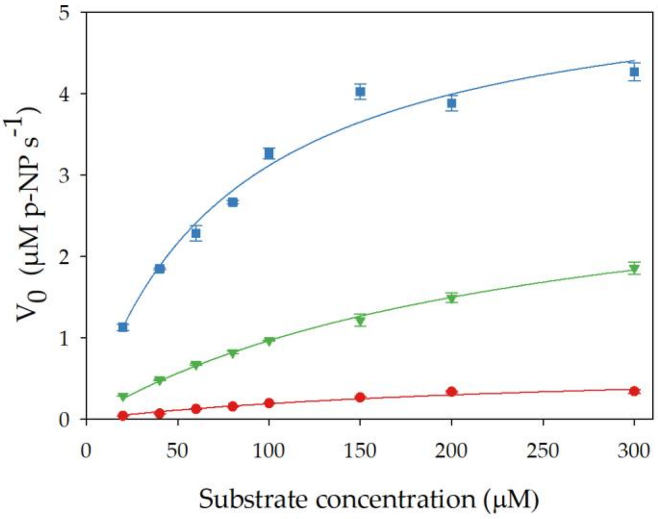

Figure S7. Enzymatic kinetics of rHsEst of the hydrolysis of *p*-nitrophenyl acetate (●), *p*-nitrophenyl valerate (■) and *p*-nitrophenyl octanoate (▾). Each point represents the average value of three independent replicates and the bars indicate the standard error. Solid lines represent the fit of Michaelis-Menten model.
